# Establishment and application of a quadruple target real-time PCR assay for detecting *Yersinia pestis*

**DOI:** 10.1371/journal.pone.0350660

**Published:** 2026-06-01

**Authors:** Wenfang Wang, Xiaoxue Zhang, Hui Yu, Zhanli Wang

**Affiliations:** 1 Inner Mongolia Key Laboratory of Disease-Related Biomarkers, The Second Affiliated Hospital, Baotou Medical College, Baotou, China; 2 School of Basic Medicine, Baotou Medical College, Baotou, China; Institut Pasteur de Madagascar, MADAGASCAR

## Abstract

In order to establish an efficient method for high-throughput detection of *Yersinia pestis (Y. pestis)*, a quadruple target real-time polymerase chain reaction assay was developed based on the specific target genes of *Y. pestis* (*caf1*, *pla*, *ymt*, and *ypo-1094*). Its sensitivity, specificity, and repeatability were evaluated, and clinical serum samples were tested by the established method. The results showed that no cross-reactivity was observed with other bacterial nucleic acids. The optimal linear detection range for *caf1*, *pla*, *ymt*, and *ypo-1094* was 12.09 × 10^-^⁶-12.09 × 10^1^ ng/μL, and the lower limit of detection was 12.09 × 10^−4^ ng/μL. Four different DNA concentrations of *caf1*, *pla*, *ymt*, and *ypo-1094* (10^−4^, 10^−3^, 10^−2^, 10^−1^and 10^1^ ng/μL) were tested five times, achieving good repeatability. In the clinical sample detection, all the *Y. pestis* – positive samples were identified. The established method has potential for clinical use for rapid detection of *Y. pestis* with high specificity and high sensitivity.

## 1. Introduction

*Y. pestis* is the etiological agent responsible for plague [1]. In natural settings, the transmission of plague predominantly occurs among wild rodent populations. Human infection typically results from flea bites carrying *Y. pestis* or through the inhalation of aerosolized bacteria. Without timely diagnosis and appropriate treatment, the disease can progress swiftly, often leading to fatal outcomes [2–4]. Historically, *Y. pestis* has been the cause of three significant pandemics, which collectively resulted in the death of tens of millions of individuals worldwide [5]. Currently, plague continues to manifest sporadically across various regions globally, with incidence rates exhibiting an increasing trend [6]. In China, plague cases are primarily reported in remote pastoral regions characterized by limited medical infrastructure and underdeveloped diagnostic and therapeutic capacities [7,8]. Healthcare providers at the primary care level frequently encounter difficulties due to inadequate professional knowledge and restricted practical expertise. Consequently, these conditions highlight the urgent need for a rapid, reliable, and field-applicable molecular diagnostic method [9].

Polymerase chain reaction (PCR) is a cornerstone molecular biology technique that enables exponential amplification of specific DNA sequences by mimicking in vitro DNA replication. In the context of *Y. pestis* detection, conventional PCR relies predominantly on sequence-specific primers designed to anneal to well-defined genomic regions, thereby facilitating targeted amplification of genes of interest. This method offers several key advantages, including operational simplicity, high analytical sensitivity, and robust specificity [10]. Consequently, conventional PCR is widely employed for *Y. pestis* identification due to its proven sensitivity and specificity. However, standard PCR assays typically amplify a single genetic locus, rendering them vulnerable to false-negative results when applied to genetically diverse or atypical strains. Notably, naturally occurring *Y. pestis* isolates may lack one or more of the species’ characteristic plasmids, thereby undermining the reliability of plasmid-based diagnostic targets [11]. As a result, single-gene assays often suffer from diminished specificity and elevated false-negative rates, particularly in differentiating *Y. pestis* from closely related *Yersinia* species [12,13]. These limitations underscore the urgent need for more resilient and comprehensive molecular detection strategies.

Multi-target PCR approaches have been proposed to enhance diagnostic reliability by enabling the concurrent detection of multiple, independent genetic markers [14–16]. Nevertheless, expanding multiplexing beyond three targets remains technically demanding due to challenges such as primer–probe cross-reactivity, disparities in amplification efficiency across targets, and spectral overlap or interference among fluorescent detection signals. Despite these constraints, quadruplex real-time PCR assays represent a promising advancement: they integrate four distinct genetic targets into a single reaction, thereby improving diagnostic confidence while simultaneously reducing assay turnaround time and operational cost. Accordingly, meticulous optimization of assay design is essential to ensure stable, specific, and quantitatively accurate multi-target detection.

To enhance detection robustness, we selected three plasmid-encoded genes (*caf1*, *pla*, and *ymt*) and one chromosomal gene (*ypo-1094*) for combined detection [17,18]. Among these genes, the plasmid-encoded genes *caf1* and *pla* are widely used as diagnostic markers due to their high specificity and sensitivity. The *caf1* gene is located on the *pMT1* plasmid and encodes the *F1* capsular antigen, which is a well-established virulence factor and a reliable indicator of the pathogenic *Y. pestis* [19]. The *pla* gene is carried by the *pPCP1* plasmid and encodes the plasminogen activator. It has been widely applied in molecular diagnostics because of its strong amplification efficiency and diagnostic sensitivity [20]. The *ymt* gene, also located on the *pMT1* plasmid, encodes the *Yersinia* murine toxin and plays a critical role in flea-borne transmission, making it a stable and epidemiologically relevant target for plague detection [21]. Inclusion of *ymt* provides an additional plasmid marker that complements *caf1* and *pla*, thereby increasing detection robustness. Furthermore, *ypo-1094* is highly conserved among *Y. pestis* strains and absent in closely related *Yersinia* species, which enables reliable species-level identification independent of plasmid presence. Therefore, the combined detection of plasmid-encoded and chromosomal targets was therefore designed to enhance both sensitivity and specificity while ensuring diagnostic reliability in genetically diverse *Y. pestis* isolates.

Here, we develop and validate a quadruple target real-time PCR assay for *Y. pestis* that simultaneously targets four distinct genetic loci. The assay undergoes rigorous evaluation for analytical specificity and sensitivity, with particular attention to minimizing false-negative results arising from plasmid loss—a known challenge in *Y. pestis* detection. Furthermore, its practical utility is assessed using simulated clinical specimens. This assay represents a robust, efficient, and reliable tool for accurate *Y. pestis* identification, with broad applicability in plague surveillance and rapid outbreak response.

## 2. Materials and methods

### 2.1 Isolation of strains

The DNA of the *Y. pestis* EV76 strain was obtained from the Inner Mongolia Autonomous Region Center for Disease Control and Prevention. The specificity of the method was assessed by testing nucleic acid from *Y. pestis*, *Y. pseudotuberculosis*, *Y. enterocolitica* strains and another 10 non-Yersinia microorganisms. These included *Escherichia coli*, *Pseudomonas aeruginosa*, *Proteus mirabilis*, *Enterobacter cloacae*, and *Lactobacillus acidophilus*. The Gram-negative bacterial strains were supplied by the Microbiology Department of the Second Affiliated Hospital of Baotou Medical College, whereas the *Y. enterocolitica* and *Y. pseudotuberculosis* strains were procured from the BeNa Culture Collection (BNCC) (Table 1).

**Table 1:** Strains and sources for multiplex PCR detection.

Strain	Numbers	Source
*Y. pestis* Ev76	1	Inner Mongolia Center for Disease Control and Prevention
*Y. enterocolitica*	1	BeNa Culture Collection
*Y. pseudotuberculosis*	1	BeNa Culture Collection
*E. coli*	2	The Second Affiliated Hospital of Baotou Medical College
*P. aeruginosa*	2	The Second Affiliated Hospital of Baotou Medical College
*P. mirabilis*	2	The Second Affiliated Hospital of Baotou Medical College
*E. cloacae*	2	The Second Affiliated Hospital of Baotou Medical College
*K. oxytoca*	2	The Second Affiliated Hospital of Baotou Medical College

### 2.2 Blood sample

Clinical blood specimens utilized in this study were collected from patients attending the Second Affiliated Hospital of Baotou Medical College from 13/09/2025–15/09/2025. Written informed consent was obtained from all participants prior to inclusion in the study. The research protocol adhered to applicable medical ethical guidelines and received approval from the Medical Ethics Committee of the Second Affiliated Hospital of Baotou Medical College (Approval No. 2025-ZX-088).

### 2.3 Bacterial cultivation and DNA extraction

The aforementioned strains were incubated overnight at 37°C in a constant temperature incubator with 5% carbon dioxide. Following the manufacturer’s protocols, bacterial genomic DNA and clinical blood DNA were extracted utilizing the bacterial genomic DNA extraction kit and the blood DNA extraction kit (Tiangen Biotech, Beijing Co., Ltd), respectively..

### 2.4 Primer design and synthesis

The *caf1*, *pla*, *ymt*, and *ypo-1094* genes of *Y. pestis* were chosen as specific target loci for the development of a quadruple-target real-time PCR assay. Primer sequences corresponding to these target genes were designed utilizing NCBI databases, informed by the published chromosomal and plasmid sequences of *Y. pestis*. These primers were subsequently synthesized by Shanghai Sangon Biotech Co., Ltd. (Shanghai, China) (Table 2).

**Table 2:** Primers employed in real-time PCR for the identification of *Y. pestis* DNA.

Gene	Sequence (5’ to 3’)	Size of PCR amplification product (bp)
*caf1*	CAGCCCGCATCACTCTTACA	102
	CGCCAAGAGTAAGCGTACCA	
*pla*	TGCAGGCCAGTATCGCATTA	131
	GTGAGCCGGATGTCTTCTCA	
*ymt*	TTCTGCTTATGGCTCCCAGC	183
	TCCTCTTGCCGTTGCTTCAT	
*ypo-1094*	CAGTAGAGCAACGGCCTCTT	101
	TTAGCACGCGCCAGAGATT	

### 2.5 Quadruple target real-time PCR

Amplification was conducted using the ABI 7500 real-time fluorescence quantitative PCR system with TaKaRa amplification reagents. The total reaction volume was 20.0 μL, comprising 10 μL of TB Green Premix Ex Taq II, 0.27 μL each of forward and reverse primers (resulting in a final primer concentration of 0.4 ng/μL), 0.4 μL of ROX Reference Dye II, 2 μL of DNA template, and 6 μL of sterilized deionized water. The thermal cycling protocol consisted of an initial denaturation at 95°C for 30 seconds, followed by 40 cycles of denaturation at 95°C for 5 seconds and annealing/extension at 64°C for 34 seconds.

In this multiplex PCR assay, non-specific amplification was observed after 30 cycles of amplification, most likely attributable to primer-dimer formation. Based on these observations, the following criteria were established for result interpretation: samples with a Ct value ≥ 30 were classified as negative; those with a Ct value < 30 were classified as positive. Samples exhibiting a Ct value < 15 were considered to contain excessively high target DNA concentrations; in such instances, sample dilution followed by repeat amplification was recommended.

### 2.6 Sensitivity analysis

The sensitivity of the assay was determined based on the limit of detection (LOD). The concentration of DNA from the *Y. pestis* EV76 strain was quantified in triplicate using a UV spectrophotometer, resulting in an average concentration of 12.09 ng/μL. Subsequently, the *Y. pestis* EV76 DNA was subjected to a series of tenfold serial dilutions with distilled water, generating seven concentration gradients ranging from 12.09 ng/μL to 12.09 × 10^−^⁶ ng/μL. DNA was then extracted sequentially from bacterial suspensions at each concentration level and amplified via real-time PCR. Throughout the experiments, distilled water was employed consistently as a negative control.

A sensitivity analysis was conducted using serial dilutions of genomic DNA. The DNA concentration was converted into genome copy numbers by considering the genome size, the average molecular weight per base pair, and Avogadro’s constant [22,23]. In brief, the genome copy number was determined using the formula:


$$\begin{array}{c} \text{Genome copies}/\mu \text{L}=(C\times \text{6}.\text{022}\times {\text{10}}^{\text{2}\text{3}})/\;(N\;\times \text{ 660}) \end{array}$$

where C is the DNA concentration in grams per microliter, N is the genome length in base pairs, 660 g/mol represents the average molecular weight of a single base pair, and 6.022 × 10^23^ is Avogadro’s number. The genome size of *Y. pestis* was estimated to be about 4.6 million base pairs. Using this calculation, DNA standards were converted into genome copies per microliter and employed to assess the assay’s analytical sensitivity [24]. This approach is commonly used in quantitative PCR research to estimate genome copy numbers from DNA mass concentrations.

### 2.7 Repetitive analysis

The DNA of the *Y. pestis* EV76 strain was serially diluted to concentrations spanning from 12.09 ng/μL to 12.09 × 10^−^⁶ ng/μL. Each dilution was subjected to real-time PCR amplification in a sequential manner. Distilled water served as the negative control, with five replicates established for this condition. The coefficient of variation (CV%) was computed from the cycle threshold (Ct) values to assess the reproducibility and stability of the *Y. pestis* detection assay. Furthermore, three repeated tests were conducted at different times and using different batches. By examining the differences between the batches and the daily variations, the reproducibility of this method was further verified.

### 2.8 Specificity analysis

*Y. enterocolitica*, *Y. pseudotuberculosis*, and additional Gram-negative bacilli with potential interference, including *Escherichia coli*, *Pseudomonas aeruginosa*, *Proteus mirabilis*, *Enterobacter cloacae*, and *Lactococcus lactis*, were selected as control strains. Detection was conducted utilizing the previously established quadruple target real-time PCR technique.

These strains were chosen because of their close genetic relationship to *Y. pestis* and their potential to affect molecular detection in clinical or environmental samples. Related Yersinia species were included to determine if the test can distinguish *Y. pestis* from genetically similar bacteria. Additionally, commonly found bacterial species were tested to assess the assay’s ability to avoid non-specific amplification when other microbes are present. This strategy allowed for a thorough evaluation of the assay’s specificity in conditions similar to actual diagnostic settings.

### 2.9 Blind test experiment

A total of 45 clinical blood specimens were randomly selected for analysis. Of these, 11 samples were artificially inoculated with *Y. pestis* DNA to simulate blood specimens from patients with clinical plague, whereas the remaining samples were maintained without treatment. Each processed blood sample was assigned a random identification number, followed by DNA extraction and subsequent real-time PCR analysis. The simulated positive samples were prepared by adding 5 µL of *Y. pestis* nucleic acid at a concentration of 12.09 ng/µL to 1 mL of blood. However, due to potential losses of *Y. pestis* nucleic acid during the extraction process, it is not possible to accurately estimate the final concentration of *Y. pestis* nucleic acid in the extracted sample.

## 3. Result

### 3.1 Sensitivity

The findings indicated that higher amounts of *Y. pestis* DNA led to earlier amplification, shown by lower Ct values. As the DNA concentration decreased, amplification occurred later, resulting in higher Ct values.

**Fig 1:**
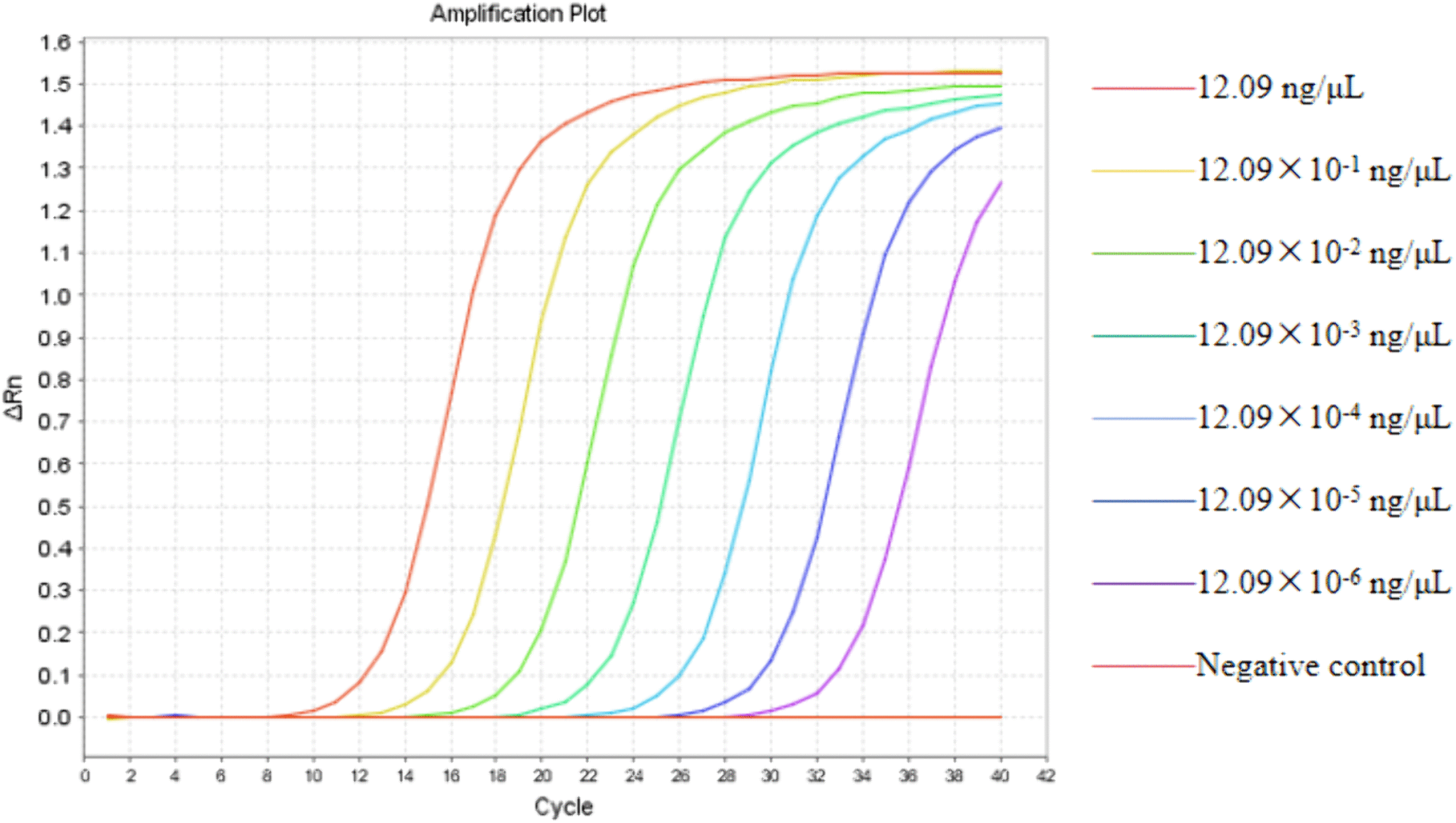
The sensitivity analysis results. The DNA concentrations of *Y. pestis* samples, arranged from left to right on the graph, range from 12.09 ng/μL to 12.09 × 10^−^⁶ ng/μL, including a negative control. The data indicate that substantial amplification was observed at sample concentrations exceeding 12.09 × 10^−4^ ng/μL.

The limit of detection (LOD) was defined as the smallest amount of target DNA that could be reliably detected in repeated tests with acceptable amplification features. A concentration was deemed detectable only if it produced a clear sigmoidal amplification curve, a Ct value within the set threshold, and 100% positive detection across replicates. Concentrations that showed inconsistent detection, greater Ct variability, or abnormal amplification curves were excluded from the LOD assessment. Using these criteria, consistent and reproducible amplification was seen at concentrations equal to or above 12.09 × 10^−^⁴ ng/µL, while lower concentrations showed less stability and reproducibility. Therefore, the LOD for the quadruple target real-time PCR assay was established at 12.09 × 10^−^⁴ ng/µL (Fig 1). This assay allows for reliable detection of low levels of *Y. pestis* DNA, facilitating early diagnosis and effective monitoring.

Since live *Y. pestis* cultures were not available in our lab, the detection limit was estimated based on DNA concentration and its conversion to genome copy number. According to the calculation in Section 2.6, the LOD corresponds to 12.09 × 10^−^⁴ ng/µL, which is approximately 240 genome copies per microliter.

### 3.2 Repeatability

**Fig 2:**
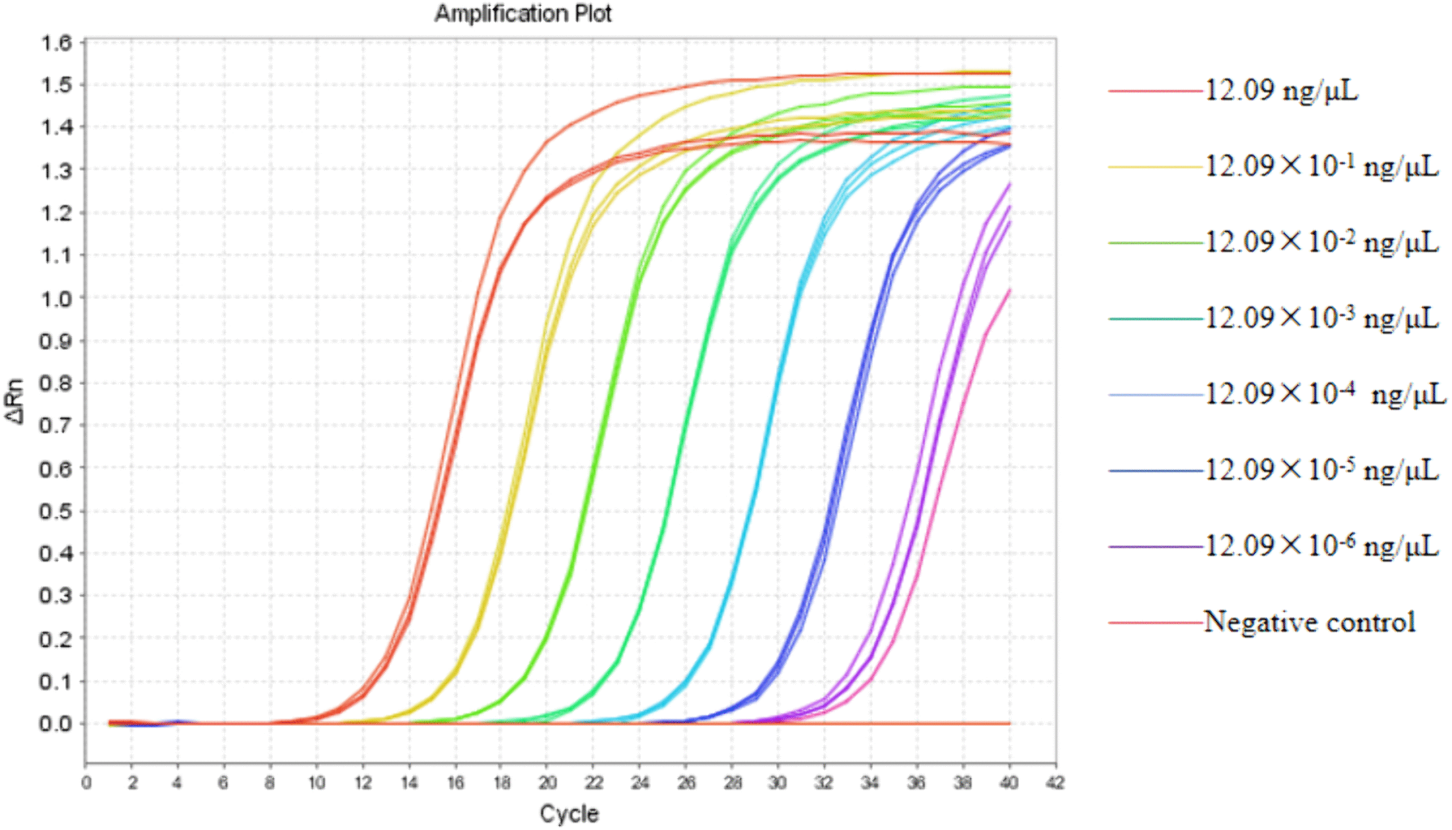
The method exhibits reproducibility at concentrations exceeding 12.09 × 10^−4^ ng/μL.

The results demonstrated that when the DNA concentration in the sample surpassed the LOD of the utilized method, the method exhibited considerable stability. Quantitative assessment showed that the CV% of the Ct values varied from 0.074% to 1.23% (Table 3). During the in-run tests and in-day tests, quantitative evaluations revealed that the coefficient of variation (CV%) of CT values was all below 5%. A standard curve was generated using a 10-fold serial dilution of the template (12.09 ng/µL). The Ct values showed a strong linear relationship with the logarithm of the template concentration across five orders of magnitude. Linear regression analysis yielded a slope of −3.7443 with an R^2^value of 0.9965, corresponding to a PCR amplification efficiency of approximately 85%.These findings confirm that the method maintains high repeatability and stability, thereby guaranteeing the reliability of the detection results (Fig 2).

**Table 3:** This table summarizes intra- and inter-batch reproducibility. Data are shown as Mean CT ± SD (n = 5 technical replicates per run; n = 3 three independent experiments).

DNA concentration (ng/μL)	Intra-assay Mean CT ± SD (n = 5)	Inter-assay Mean CT ± SD (n = 3)
12.09	1.33 ± 0.16	13.39 ± 0.22
12.09 × 10^−1^	16.67 ± 0.13	16.67 ± 0.17
12.09 × 10^−2^	20.67 ± 0.10	20.67 ± 0.17
12.09 × 10^−3^	24.89 ± 0.25	24.86 ± 0.13
12.09 × 10^−4^	28.97 ± 0.3	28.76 ± 0.18

### 3.3 Specificity

**Fig 3:**
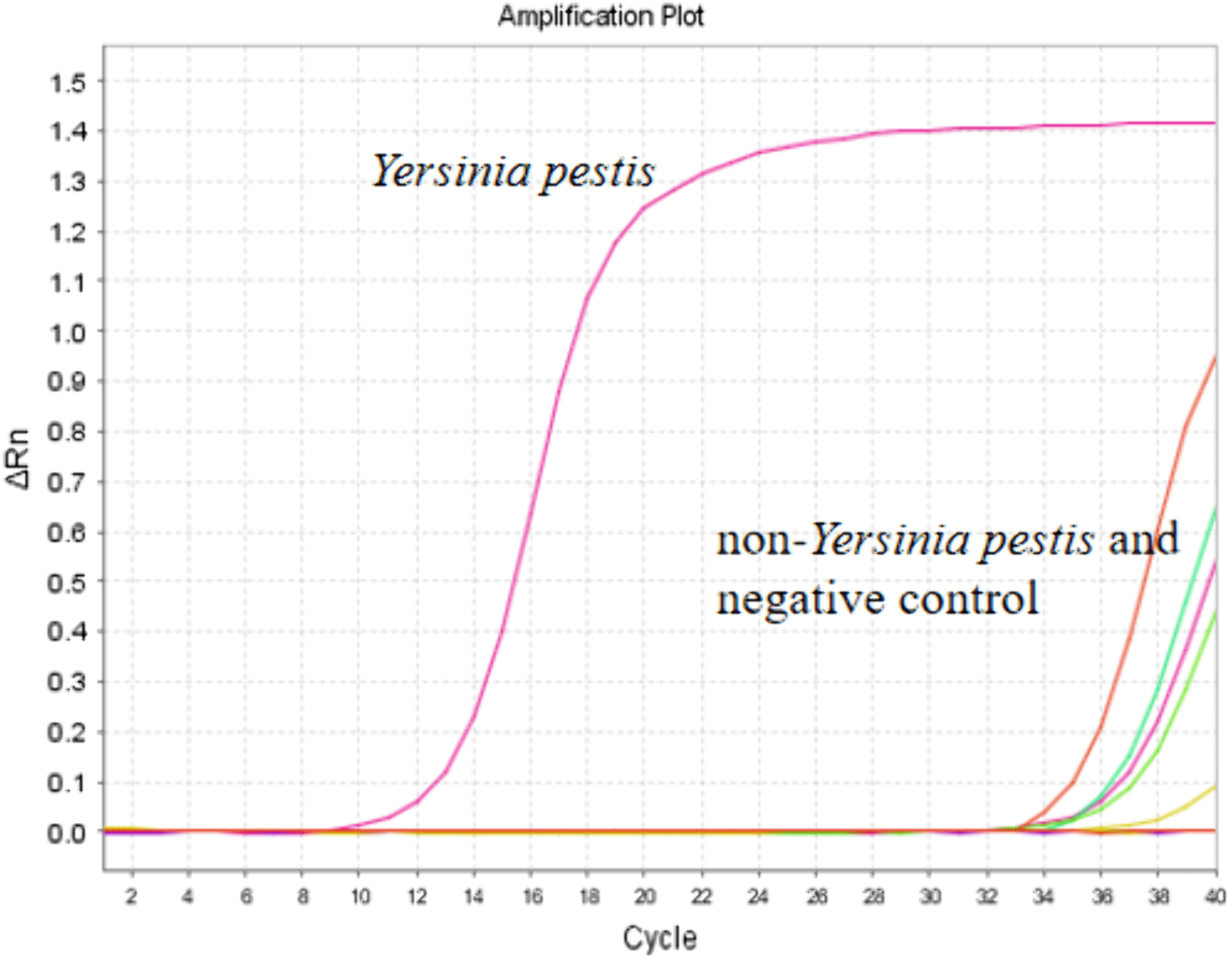
The results of the specificity analysis indicated that only *Y. pestis* tested positive.

The evaluation results indicated that the established method exhibits remarkably high specificity solely for *Y. pestis*. It attained a specificity rate of 100% and showed no cross-reactivity with other bacterial species. However, non-specific amplification was detected after the 30th cycle, which was attributed to the formation of primer dimers (Fig 3)

### 3.4 Blind test

**Fig 4:**
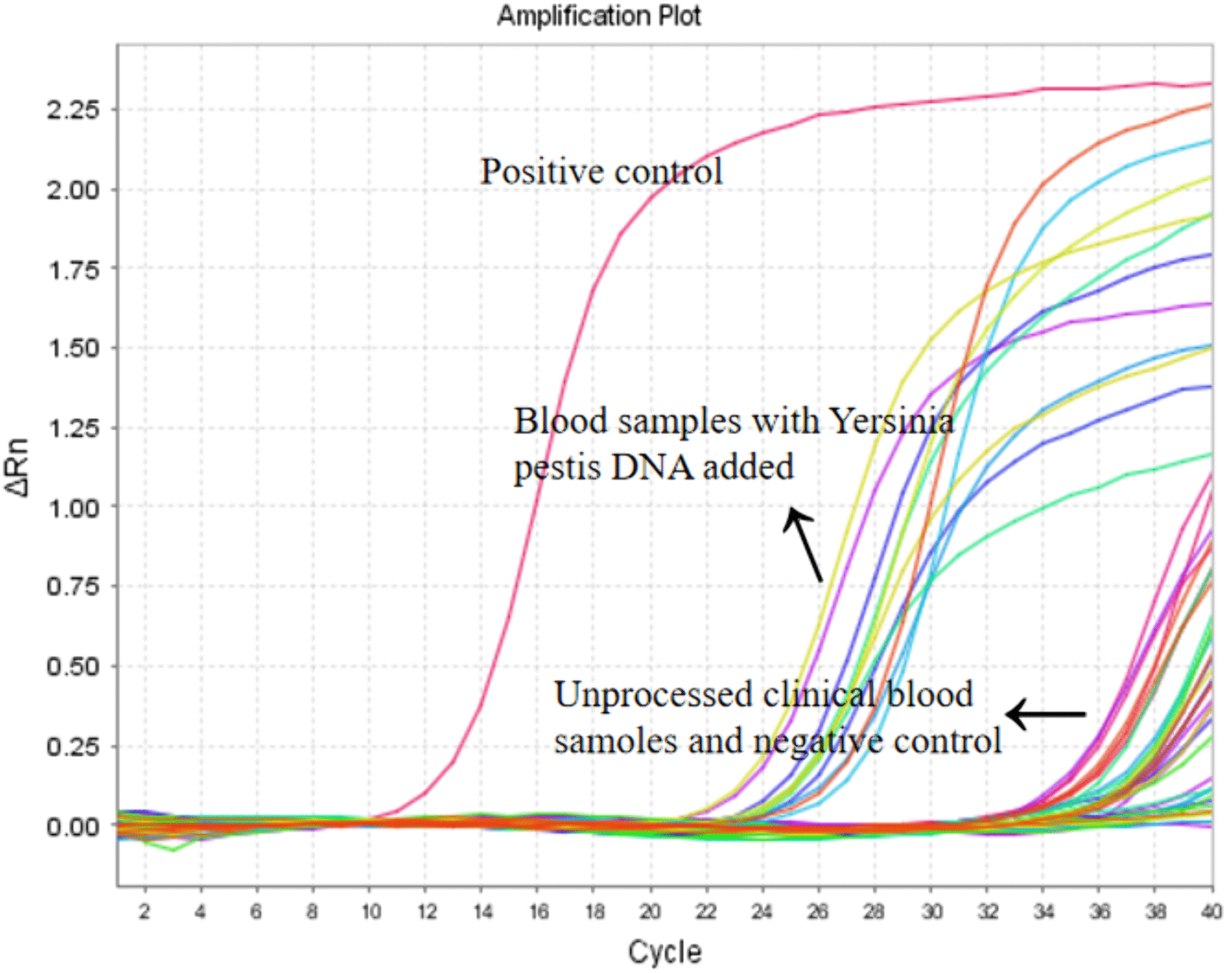
The results demonstrated that specific amplification occurred in 11 clinical blood samples that had been artificially inoculated with *Y. pestis* DNA to mimic blood specimens from patients diagnosed with clinical plague. No specific amplification was found in other samples.

The findings indicated that targeted amplification was observed in 11 clinical blood samples that had been artificially inoculated with *Y. pestis* DNA to simulate blood specimens from patients diagnosed with clinical plague. The CT values of the samples were all less than 30. No targeted amplification was detected in the remaining samples. Their CT values are all greater than 30. These findings provide additional evidence that the method can consistently detect *Y. pestis* in practical applications and may represent a reliable technical approach for the diagnosis and surveillance of plague (Fig 4).

## 4. Discussion

This study aimed to develop and validate a quadruple target real-time PCR assay for the detection of *Y. pestis*. This approach incorporates four target genes—*caf1*, *pla*, *ymt*, and *ypo-1094*—within a multiplex PCR framework. Prior PCR-based assays have primarily targeted the *caf1* and *pla* genes [25–27]. Although these genes exhibited high specificity, atypical strains lacking one or more plasmids have been reported in natural settings. In addition, the *pla* gene has been identified in other bacterial species, raising concerns regarding assay specificity. These limitations indicate that single-target assays are susceptible to both false-negative and false-positive results. The present strategy was therefore designed to improve diagnostic accuracy and robustness through multi-target detection.

The assay demonstrated strong specificity for *Y. pestis*, showing no cross-reactivity with the other bacterial species tested. Detecting four target genes simultaneously lowers the chance of false-negative results caused by plasmid loss, thereby enhancing diagnostic accuracy.

The LOD was found to be 12.09 × 10^−^⁴ ng/µL, which corresponds to about 240 genome copies per microliter. This level of sensitivity aligns with previously reported PCR-based assays for *Y. pestis* [28]. While some single-target assays may achieve slightly better detection limits under ideal conditions, this method remains competitive, especially given the added complexity of multiplex detection. Importantly, targeting multiple genes increases resilience against genetic variations and plasmid loss, offering a practical benefit for reliable detection.

Furthermore, the assay’s sensitivity is consistent with other molecular tests for Y. pestis. Real-time PCR assays aimed at plasmid or chromosomal genes typically detect between 10 and 100 genome copies per microliter under optimized conditions [29]. Similarly, pulse-controlled amplification (PCA) assays report detection limits around 100–1,000 copies per reaction [30]. More recently, droplet digital PCR (ddPCR) has shown significantly higher sensitivity, with detection limits ranging from roughly 6–15 copies per reaction [31].

Overall, these findings suggest that PCR-based methods for detecting *Y. pestis* generally have sensitivities between 10 and 1,000 genome copies. The assay developed here falls within this range and is comparable to standard real-time PCR techniques. Although it does not reach the superior sensitivity of advanced platforms like ddPCR, it offers a balanced combination of sensitivity, specificity, and multiplexing ability. Notably, including four separate genetic targets improves detection reliability and minimizes the risk of false negatives due to plasmid loss or genetic variation, making it well-suited for routine diagnostic use.

One of the significant characteristics of plague is its sporadic nature, and it is often difficult to detect in the early stages of infection. This is particularly true for pneumonic plague, where the disease progresses rapidly, and the mortality rate is high. Early diagnosis and timely treatment are critical to reducing mortality. Therefore, there is an urgent need to develop simple and rapid diagnostic tools that can be used for initial screening in the absence of complex laboratory setups. Based on this need, we have developed a quadruple target PCR method aimed at facilitating preliminary screening of *Y. pestis* at the grassroots level. In the case of suspected results, we recommend confirming the diagnosis using the gold standard method or by employing PCR targeting two different genetic loci for validation.

To further elucidate the clinical applicability of this approach, the quadruple target real-time PCR assay was subjected to validation via a blinded experimental trial. The outcomes of the experiment aligned with the anticipated results, as significant amplification was observed exclusively in 11 DNA samples and the positive control. These findings provide additional confirmation that our method, serving as an auxiliary diagnostic tool, possesses substantial potential for clinical application, plays a critical role in the diagnostic process, and offers considerable value.

We acknowledge the limitation regarding the choice of blood as the sole clinical specimen for molecular detection of *Y. pestis*. While blood is often used in early-stage diagnostics due to its accessibility and utility in detecting systemic infection, it may not be the most reliable specimen for detecting *Y. pestis*, particularly in cases of bubonic plague, where the bacterium is more concentrated in lymphatic tissues such as the lymph nodes. Therefore, we should conduct further studies involving other clinical samples (such as adenoma samples or sputum samples, etc.) to comprehensively evaluate the applicability of this method in different clinical manifestations of plague.We consider this as a limitation in our study, and future research will aim to address this by expanding the sample types used for validation.

Given the highly infectious nature of *Y. pestis*, procedures such as bacterial culture and nucleic acid extraction must be performed within a biosafety level 3 (BSL-3) laboratory. However, due to the lack of appropriate facilities in our laboratory to handle such pathogenic microorganisms, this study did not include bacterial culture experiments. This limitation precludes the determination of the LOD at the bacterial cell level, representing a primary constraint of the current investigation. We acknowledge this shortcoming with regret. Should access to a suitable experimental platform become available in the future, we intend to prioritize the supplementation and refinement of this aspect of the study, thereby further enriching the evaluation of the proposed methodology. In addition, due to the limited availability of clinical cases and the sporadic occurrence of plague, as well as the constraints of laboratory resources, this study was conducted as a methodological validation rather than involving clinical samples. While the proposed method showed promising results in controlled experimental conditions, its performance in real-world clinical settings remains to be fully validated. We acknowledge that the lack of testing with actual clinical samples is a limitation of this study, and we recommend that future work focus on evaluating the method in clinical settings once suitable samples become available.

In general, traditional bacterial culture typically requires 48–72 hours to yield results, while the quadruple-target real-time PCR approach completes the process within 3–4 hours, encompassing both nucleic acid extraction and PCR amplification [32–34]. This substantial reduction in time facilitates more timely clinical diagnosis and intervention. Additionally, the technology’s ease of use allows laboratory staff to quickly understand and apply the method, making it more appropriate for on-site detection. Given its advantages of rapid detection and operational simplicity, this technology represents an effective tool for plague surveillance and epidemic management.

In comparison to conventional PCR methods, the quadruple target real-time PCR for *Y. pestis* enables the simultaneous amplification of multiple loci within a single PCR reaction. This technique not only conserves reagents and sample material but also reduces overall costs. The quadruple target real-time PCR developed in this study is distinguished by its capacity to concurrently detect four specific target genes—*caf1*, *pla*, *ymt*, and *ypo-1094*—demonstrating high sensitivity, specificity, and rapid diagnostic performance. These attributes render it a valuable tool for enhancing the molecular diagnosis of plague. Nonetheless, because multiplex PCR involves multiple primer pairs reacting within a single system, optimization of reaction parameters such as annealing temperature, template DNA concentration, and cycle number is essential to minimize non-specific amplification. Consequently, more precise control of experimental conditions is required, which increases the complexity of the procedure. Additionally, primer accumulation may contribute to non-specific amplification, potentially resulting in false-positive outcomes. Current challenges include issues related to DNA extraction quality and non-specific amplification. Future research should prioritize addressing these limitations to improve assay reliability and accuracy.

## Supporting information

S1 Table Sensitivity results. (XLSX)

S2 Table Repeatability results. (XLSX)

S3 Table Specificity results. (XLSX)

S4 Table Blind test results. (XLSX)
